# Shared hotspot mutations in oncogenes position dogs as an unparalleled comparative model for precision therapeutics

**DOI:** 10.1038/s41598-023-37505-2

**Published:** 2023-07-06

**Authors:** Lucas Rodrigues, Joshua Watson, Yuan Feng, Benjamin Lewis, Garrett Harvey, Gerald Post, Kate Megquier, Michelle E. White, Lindsay Lambert, Aubrey Miller, Christina Lopes, Shaying Zhao

**Affiliations:** 1 One Health Company, Inc, 530 Lytton Ave, 2nd Floor, Palo Alto, CA 94301 USA; 2 Department of Biochemistry and Molecular Biology, Institute of Bioinformatics, University of Georgia, B304B Life Sciences Building, 120 Green Street, Athens, GA 30602-7229 USA; 3 Broad Institute of MIT and Harvard, Cambridge, MA 02142 USA

**Keywords:** Cancer, Cancer genomics, Cancer models

## Abstract

Naturally occurring canine cancers have remarkable similarities to their human counterparts. To better understand these similarities, we investigated 671 client-owned dogs from 96 breeds with 23 common tumor types, including those whose mutation profile are unknown (anal sac carcinoma and neuroendocrine carcinoma) or understudied (thyroid carcinoma, soft tissue sarcoma and hepatocellular carcinoma). We discovered mutations in 50 well-established oncogenes and tumor suppressors, and compared them to those reported in human cancers. As in human cancer, *TP53* is the most commonly mutated gene, detected in 22.5% of canine tumors overall. Canine tumors share mutational hotspots with human tumors in oncogenes including *PIK3CA*, *KRAS*, *NRAS*, *BRAF*, *KIT* and *EGFR*. Hotspot mutations with significant association to tumor type include NRAS G61R and PIK3CA H1047R in hemangiosarcoma, ERBB2 V659E in pulmonary carcinoma, and BRAF V588E (equivalent of V600E in humans) in urothelial carcinoma. Our findings better position canines as a translational model of human cancer to investigate a wide spectrum of targeted therapies.

## Introduction

New approaches to the development of cancer therapeutics are urgently needed to improve the current 89% failure rate of novel drugs in clinical trials^1–3^, and to improve patient outcomes. Spontaneous cancer in companion animals represents a unique opportunity for investigation of novel therapeutics for human and veterinary use^4–15^. Dogs develop spontaneous tumors that are highly similar to human cancers in terms of histological features and clinical presentation, but canine tumors typically progress more rapidly^7,10,11,16^. Canine cancers have also been found to have similar genetic and molecular targets to human malignancies^8,10,17–30^, and thus affected dogs present an opportunity to test novel therapeutics in a treatment-naive setting that is not currently feasible in human medicine^11^. Dogs represent a large animal model with an intact immune system, enabling comparative studies of therapeutic efficacy, immunotherapy, tumor evolution, and tumor microenvironment^7,11,31^. Studies of new therapeutic agents have begun to include dogs with cancer to help characterize pharmacokinetic and pharmacodynamic properties, efficacy, and tolerability^11,31^. In addition, the National Cancer Institute’s Center for Cancer Research has founded the Comparative Oncology Program and the Canine Oncology Trials Consortium to support comparative studies in dogs and facilitate integration of these findings with human oncology efforts^31^.

Canine tumors provide a powerful platform for translational investigation^11,12,32^. Over the past decade, genomic characterization of canine cancers has highlighted the marked biological and molecular similarities between several canine and human cancers, including lymphoma^17,18,33^, osteosarcoma^8,23,25^, hemangiosarcoma^20,26–28,30,34^, glioma^29^, melanoma^22^, mammary tumors^35–37^, and urothelial carcinoma^19,21^. Some of the somatic mutations identified in these canine cancers occur at the orthologous position to known mutational hotspots found in human cancers, including *PIK3CA H1047*^20,26^, *BRAF V588* (V600 for human)^19^, and *FBXW7 R470* (R465 for human)^18^. These somatic mutations do not always occur in the same cancer type across species, more canine studies are needed to better characterize this association.

Although studies have shown genomic concordance between canine and human cancers, the number of canine tumors that have undergone genomic sequencing lags behind human tumors by an order of magnitude (fewer than 2000 canine tumors have been sequenced^38^, compared to more than 20,000 human tumors^39^). Consequently, the landscape of actionable tumor mutations in canine cancers is not fully understood^6,25^. We sought to address this issue in order to assess the feasibility of matching dogs with spontaneous cancers to targeted therapy, thereby providing treatment opportunities to canine patients while developing a platform that could accelerate a more global understanding of the clinical as well as translational potential from dogs to humans.

To do this, we developed a next-generation sequencing (NGS) panel targeting coding exons of 59 genes frequently mutated in human cancers. Using this panel, we performed the largest sequencing study of canine cancers to date, including 671 tumors of 23 histologic types from dogs representing more than 96 breeds. Importantly, our study revealed 18 canine mutational hotspots, 8 of which were orthologous to hotspots reported in human cancers and clinically actionable. These results demonstrate significant overlap in somatic hotspot mutations between human and canine cancers, further highlighting spontaneous canine cancers as an excellent model for the investigation of targeted therapies.

## Results

### Cohort demographics

**Figure 1:**
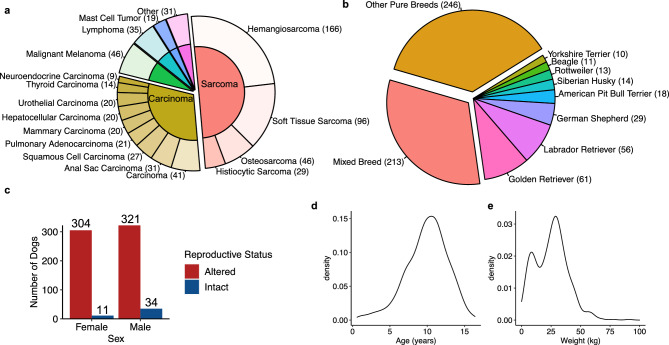
Demographics of enrolled 671 dogs. (**a**) Distribution of tumor supertypes (inner cycles) and types (outer circles). The numbers inside the parentheses indicate the dog numbers. The locations of the 41 carcinomas are provided in Table S1. (**b**) Distribution of breed. Breeds with ≥ 10 dogs are specified. (**c**) Distribution of sex and reproductive status. (**d**) Distribution of age. (**e**) Distribution of weight.

The cohort consisted of 671 dogs with tumors representing 23 cancer types. Hemangiosarcoma are the most common tumor type (n = 166), followed by soft tissue sarcoma (n = 96), melanoma (n = 46), osteosarcoma (n = 46), lymphoma (n = 35) and anal sac carcinoma (n = 31) (Fig. 1a). In total, 337 sarcomas, 203 carcinomas, and 131 other cancer types were included (Fig. 1a; Table S1). For each case of mammary carcinoma, lymphoma and mast cell tumor, we extracted the tumor subtype and grade data available from the original histopathological reports and provided the data in the Table S1.

The cohort consisted of both purebred (n = 457 dogs, 96 breeds), and mixed breed (n = 213, ≥ 45 breeds) ancestry dogs (Table S1) as reported by owner. A total of 9 breeds are represented by ≥ 10 dogs (Fig. 1b; Table S1). The largest breed groups are Golden Retrievers (61 pure and 17 mixed), Labrador Retrievers (56 pure and 29 mixed), German Shepherd Dogs (29 pure and 2 mixed), and American Pit Bull Terriers (20 pure and 15 mixed) (Fig. 1b). A total of 355 cases are male (321 neutered and 34 intact) and 315 are female (304 spayed, 11 intact, and 1 unknown) (Fig. 1c; Table S1). Dogs ranged in age from 1 to 16 years (mean 9.9 ± 2.8) (Fig. 1d, Table S1). Interestingly, dogs with osteosarcoma are significantly younger than dogs with other tumor types, with an average age of 8.4 years compared to 10.1 years respectively (Fig. S1). Weights ranged from 1 to 91 kg (mean 25.5 ± 13.5) (Fig. 1e; Table S1).

### Germline-somatic mutation discrimination

**Figure 2:**
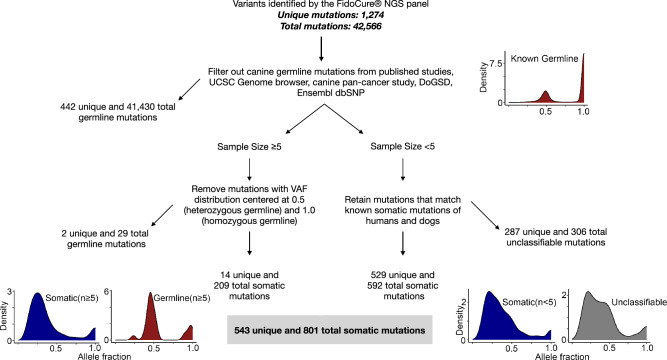
Germline-somatic mutation discrimination pipeline. The pipeline first filtered out known germline mutations reported in literatures and databases, or identified in normal samples. The remaining mutations were then divided into two groups. For mutations identified ≥ 5 dogs, variant allele fraction (VAF) distribution of each mutation was examined to determine if the mutation is germline (clustered at 0.5 for heterozygous germline and near 100% for homozygous germline) or somatic mutation (random distribution). For mutations identified in < 5 dogs, a mutation would be classified as somatic if its human orthologous mutation was found in COSMIC or cBioPortal (both databases host human somatic mutations). Total and unique mutation numbers, along with VAF distributions, were shown for each major step.

The 671 tumors were subjected to targeted sequencing of the FidoCure^®^ NGS panel, which contains 59 oncogenes and tumor suppressors frequently mutated in human cancer. A total of 42,566 mutations (1274 unique mutations) were called by comparing the sequences to the CanFam3.1 genome. These mutations, however, consisted of both germline and somatic mutations. As no normal samples from these dogs were sequenced, we developed a pipeline for germline-somatic mutation discrimination based on known canine germline mutations, variant allele frequency (VAF) distribution, and known human somatic mutations (Fig. 2). Briefly, we first filtered out germline variants published in various databases and literature (see “Methods”)^38,40–43^. As a result, 41,430 total (442 unique) mutations were classified as germline variants and were excluded (Fig. 2). We then divided the remaining mutations into two groups, based on their rate of recurrence. For mutations found in ≥ 5 dogs, we examined the VAFs. Mutations with a VAF distribution clustered around 50% or near 100% were classified as heterozygous or homozygous germline mutations respectively. As a result, 29 total mutations (2 unique) mutations were excluded (Fig. 2). For mutations found in < 5 dogs, we identified those for which the human counterparts are known somatic mutations in human cancers, and considered them somatic mutations (Table S2). As a result, 592 total (529 unique) mutations were classified as somatic (Fig. 2), with the remaining 306 total (287 unique) mutations with the germline/somatic status unknown (unclassifiable in Fig. 2).

**Figure 3:**
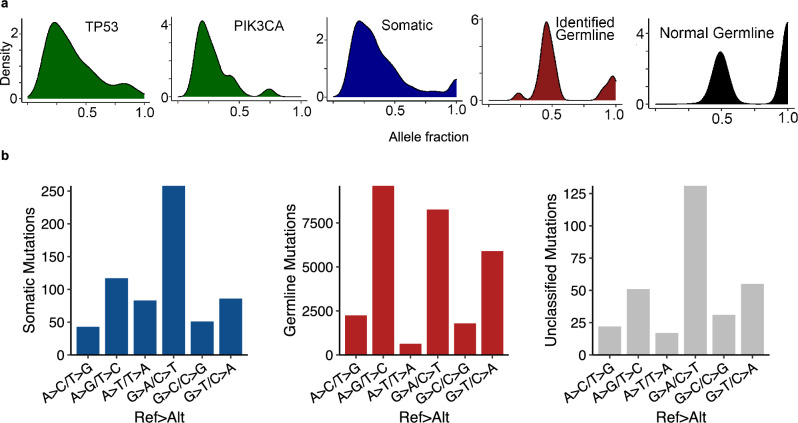
Identified germline-somatic mutation comparison. (**a**) VAF distributions of germline mutations and somatic mutations. *TP53* and *PIK3CA* mutations are all somatic (via manual examination). The VAF distribution of somatic mutations identified by Fig. 2, along with the VAF distribution of mutations detected in 20 normal samples sequenced, are also shown. (**b**) The distribution of the 6 base substitution types of somatic, germline and unclassified mutations identified from Fig. 2.

Our pipeline classified 801 total (543 unique) mutations as somatic (Fig. 2). The VAF distribution resembled those of known somatic mutations (p > = 0.32), including *TP53* and *PIK3CA* mutations, but differed from those of known and identified germline mutations in these tumor samples (p < 1 × 10^−6^) (Fig. 3a). Importantly, we repeated the same sequencing and GATK mutation calling to samples from 20 healthy dogs, collected using cheek swabs. The VAF distribution of these normal samples resembled germline mutations (p > 0.99), but not somatic mutations (p < 1 × 10^−15^), found in the tumor samples (Fig. 3a). Importantly, among 411 unique variants called in normal samples and 546 unique somatic mutations identified by our pipeline (Fig. 2) in tumors samples, only 6 (1%) mutations were shared (Table S2). Lastly, the identified somatic mutations had a base substitute type pattern matching that of somatic mutations, but not germline mutations (Fig. 3b), e.g., G > A/C > T mutations being dominant. These observations indicate that our pipeline (Fig. 2) is effective.

### Somatic mutational landscape

**Figure 4:**
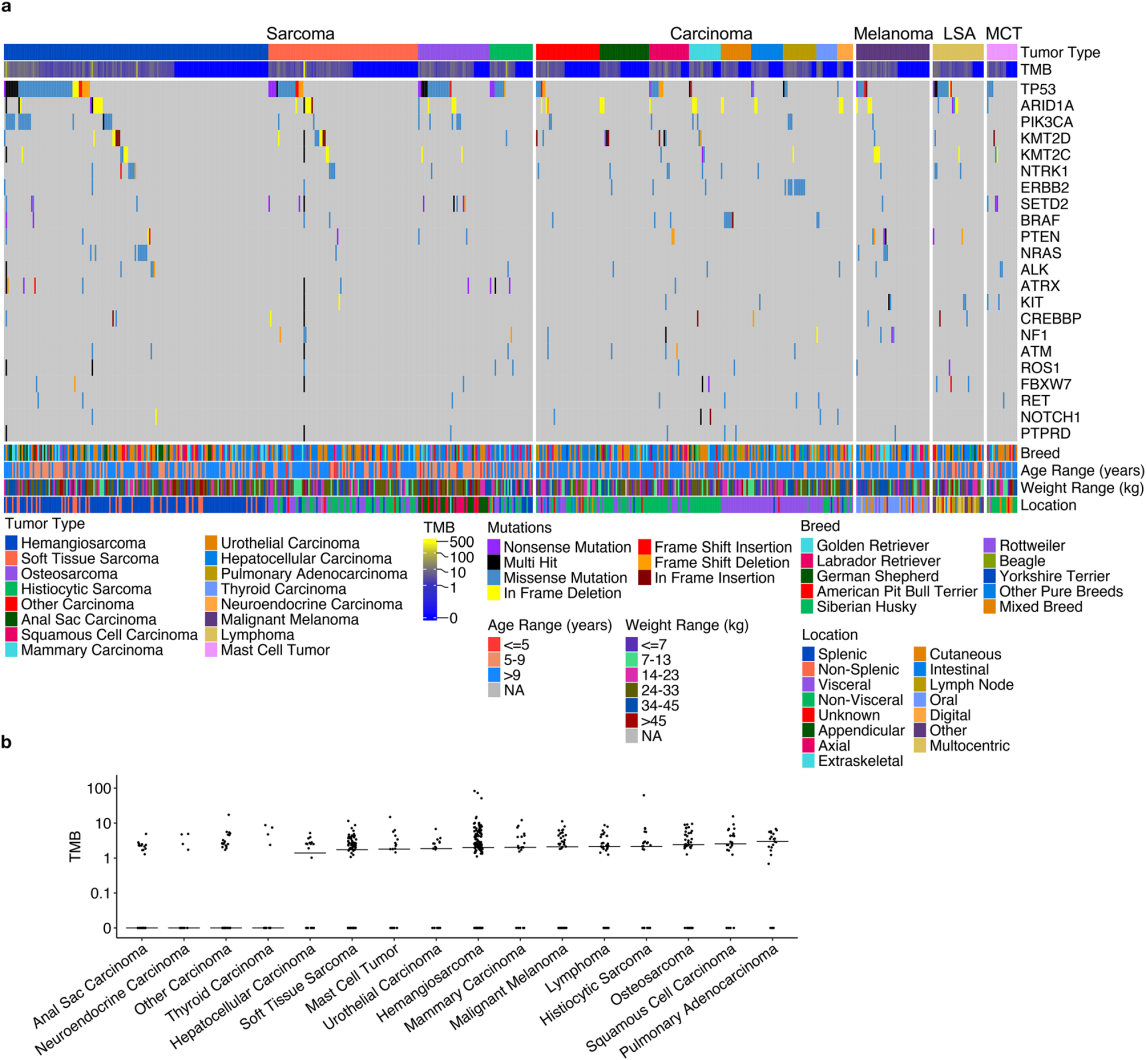
Somatic mutational landscape in canine tumors. (**a**) Oncoprint of the 22 most frequently mutated genes across the cohorts in each tumor sample, grouped by tumor supertype (sarcoma, carcinoma, and other indicated at the top), tumor types, and mutations. Tumor mutation burden (TMB), breed, age, weight and tumor location are also indicated. *LSA* lymphoma, *MCT* mast cell tumor. (**b**) Distribution of TMB in each tumor type. Each dot represents a sample, while the black horizontal lines indicate the median TMB in the respective cancer types.

Many of the somatic mutations discovered by our pipeline are consistent with published studies^18,20–23,25,26,29,38^. For example, *TP53* is the most frequently mutated gene across the cohort, mutated in 151 out of 671 animals (22.5%) (Fig. 4a). *PIK3CA* is the third most mutated gene across the tumor types, with the mutation especially common in hemangiosarcoma, mutated in 13% of the samples (Fig. 4a; Table S2). *ERBB2* and *BRAF* are the most frequently mutated genes in pulmonary adenocarcinoma and urothelial carcinoma, mutated in 50% and 40% of the samples respectively (Fig. 4a).

Our pipeline identified known mutational hotspots. These included *PIK3CA* H1047R (n = 16), *ERBB2* V659E (n = 13), *BRAF* V588E (n = 13), *NRAS* Q61R (n = 6), *NRAS Q61K* (n = 6), *TP53* R209H and R226H (n = 6 each) (Table S2). These mutations are known or likely cancer drivers, and most of them are activating or gain-of-function changes.

Our study revealed findings not previously reported. For example, chromatin remodeler *ARID1A* is the second most frequently mutated gene of the cohort, mutated across tumor types at a rate of 8% (Fig. 4a). Moreover, about 78% (40/51) of mutations in *ARID1A* are in-frame deletions (Fig. 4a; Table S2), the significance of which remains to be determined. One deletion, *ARID1A* 1038_1040del variant was called in one normal sample (Table S2), likely because it locates in a GCC repetitive region and has a higher chance of being deleted. The increased frequency of *ARID1A* 1038_1040del in tumor samples may arise from the increased instability of tumor genomes. Other chromatin modeler genes *KMT2D*, *KMT2C*, *SETD2*, and *CREBBP* are the third, fourth, seventh, eighth and fifteenth most mutated genes across the cohort (Fig. 4a). Those mutations appear to be randomly distributed among the tumor supertypes and types (Fig. 4a). *SETD2* is mutated in 18 tumors, 7 of which harbored *SETD2* truncation mutations, consistent with previous findings of frequent truncation mutation in *SETD2*^1,2^.

Our study provided a snapshot of somatic mutations for > 9 canine carcinomas, including previously uncharacterized tumor types such as anal sac carcinoma and neuroendocrine carcinoma, as well as less characterized tumor types including hepatocellular carcinoma and thyroid carcinoma (Fig. 4). These four types of carcinomas appear to have different mutational landscapes than other carcinomas, including depletion of *TP53* mutation (Fig. 4a). The mutations also appear more random, lacking a prominent mutated gene like *ERBB2* in pulmonary cancer and *BRAF* in bladder cancer (Fig. 4a). Lastly, they also have lower TMB values overall for genes included in the targeted panel (Fig. 4b).

Other previously less-characterized tumor types include soft tissue sarcoma, histiocytic sarcoma, and mast cell tumor. Our study also provided a more comprehensive mutation landscape for these tumors. The same as in other sarcomas, *TP53* is the most frequently mutated gene in soft tissue sarcoma and histiocytic sarcoma, mutated at 22% (21/96) and 35% (10/29) respectively (Fig. 4a). However, soft tissue sarcoma also harbors more mutations in chromatin modeler genes (e.g., *ARID1A* and *KMT2D*) and the neurotrophic tyrosine kinase receptor gene *NTR1*, as well as higher panel-specific TMB, compared to histiocytic sarcoma (Fig. 4). *TP53* is also the most frequently mutated gene in mast cell tumors, mutated in 21% (4/19) (Fig. 4a). However, in mast cell tumors, mutations of other genes are relatively rare (Fig. 4a), and the panel-specific TMB was lower overall (Fig. 4b).

### Location-specific mutation in hemangiosarcoma

**Figure 5:**
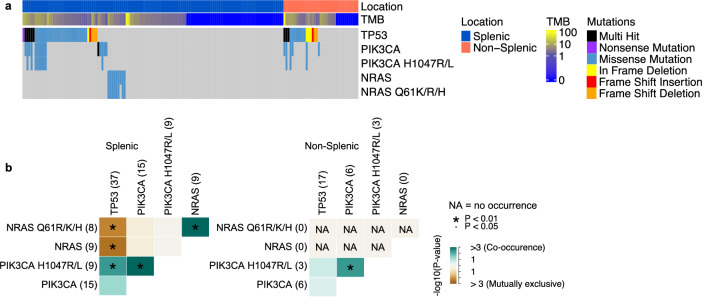
Location-specific mutation and mutually exclusive mutations in canine hemangiosarcoma. (**a**) Oncoprint of gene and specific mutations indicated in hemangiosarcomas grouped by splenic and non-splenic locations and then ordered by mutation type. (**b**) Heatmap indicating co-occurrence (green) or mutual exclusion (brown) between mutations shown in splenic and non-splenic hemangiosarcoma.

The hemangiosarcoma samples in our study consisted of 129 tumors from the spleen and 37 tumors from non-splenic locations (Fig. 5a; Table S3). *TP53* is the most frequently mutated gene in both splenic and non-splenic hemangiosarcoma, mutated at 29% (37/129) and 46% (17/37) respectively. *PIK3CA* mutations, ≥ 50% of which are H1047R/L, are also common, mutated at 12% (15/129) and 16% (6/37) in splenic and non-splenic hemangiosarcoma, respectively (Fig. 5a). However, while *NRAS* mutations, 89% of which are Q61R/K/H, are frequent in splenic hemangiosarcoma (mutated in 7% [9/129]), *NRAS* mutations are not detected in non-splenic hemangiosarcoma (Fig. 5a). Furthermore, in splenic hemangiosarcoma, *NRAS* mutations are mutually exclusive with *TP53* mutations (*p* < 0.01) and *PIK3CA* mutations (not significant, likely due to small sample size) (Fig. 5b), consistent with a previous study^3^.

### Somatic mutation enrichment and depletion

**Figure 6:**
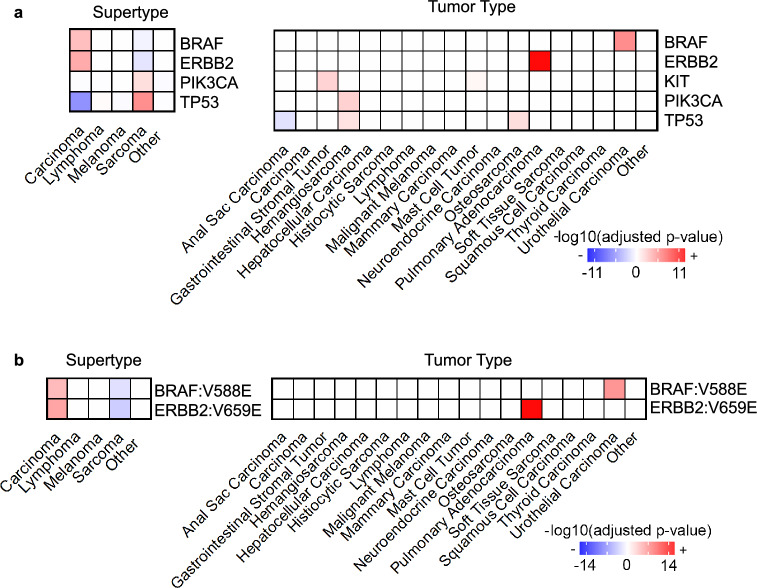
Canine somatic mutation enrichment and depletion across tumor supertype and tumor types. (**a**) Heatmaps indicating the enrichment (red) or depletion (blue) scores, based on Fisher’s exact test, of genes mutated in > 5 dogs in tumor super type or tumor type with > 20 samples. (**b**) Heatmaps for individual gene mutation, presented as described above.

Consistent with previous findings^4^, our study indicates that canine cancer mutations were tumor type dependent, but largely breed independent (Table S4). Specifically, *TP53* mutations are significantly enriched in sarcomas (p = 1.00 × 10^−7^), including hemangiosarcoma (p = 5.82 × 10^−6^) and osteosarcoma (p = 2.92 × 10^−2^), but depleted in carcinoma (p = 2.58 × 10^−7^), including anal sac carcinoma (p = 5.73 × 10^−4^) (Fig. 6a). *PIK3CA* mutation is also enriched in sarcoma including hemangiosarcoma (p = 4.70 × 10^−5^) (Fig. 6a). *BRAF* and *ERBB2* mutations, however, specifically *BRAF* V558E and *ERRB2* V659E, are depleted in sarcoma (Fig. 6a,b). These mutations are enriched in carcinoma, with significant enrichment of *BRAF* V558E in urothelial carcinoma (p = 3.92 × 10^−14^) and *BRAF* V695E in pulmonary adenocarcinoma (p = 3.52 × 10^−8^) (Fig. 6a,b). Interestingly, KIT mutation is enriched in gastrointestinal stromal tumors (p = 7.28 × 10^−5^) (Fig. 6a). We did not observe significant enrichment and depletion of somatic mutations in specific age or weight groups (Fig. 4a; Table S3).

### Comparison of dog–human mutational hotspots

**Figure 7:**
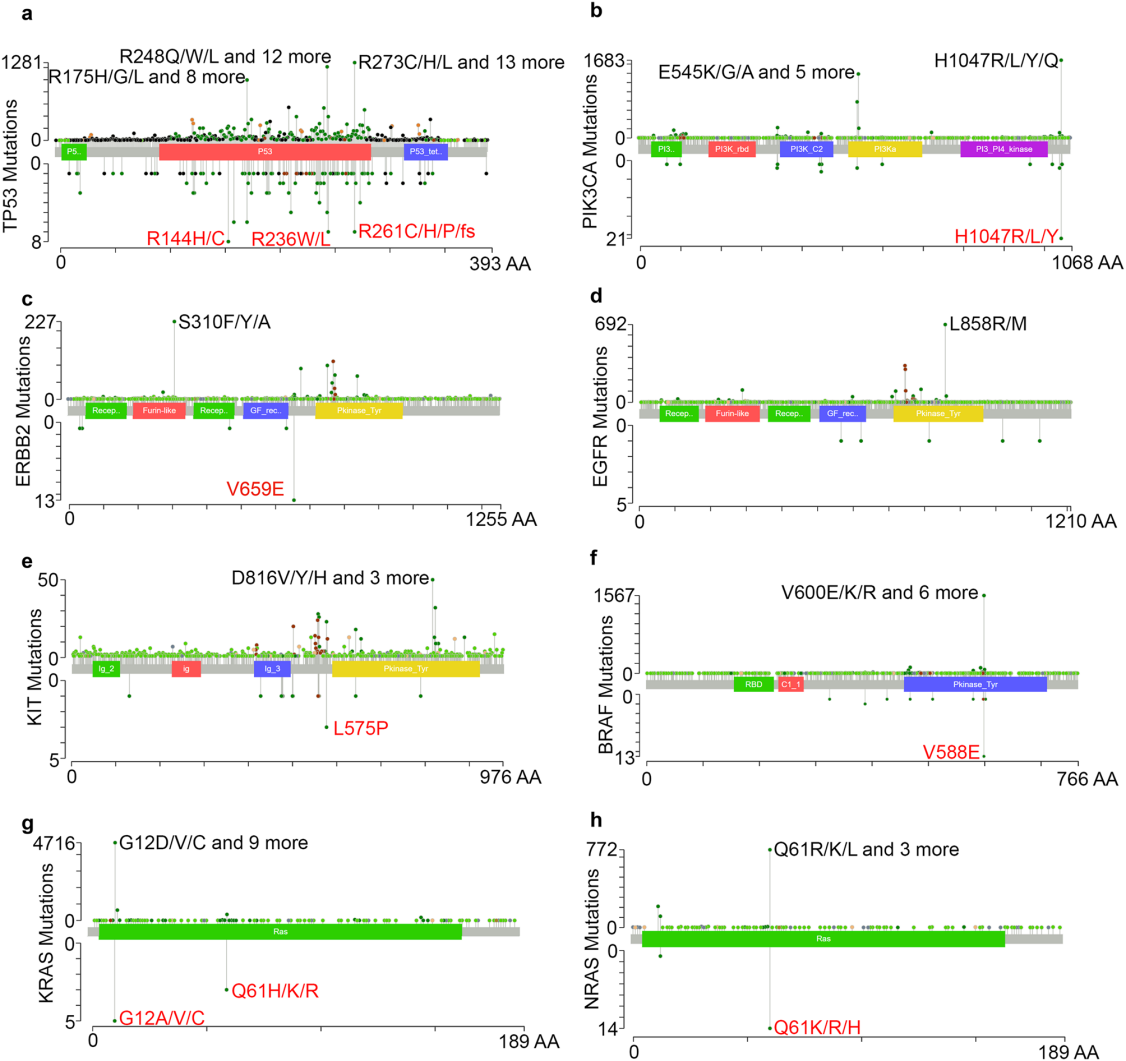
Comparison of canine and human mutational hotspots. Lollipop plots depict the mutational distribution in TP53 (**a**), PIK3CA (**b**), ERBB2 (**c**), EGFR (**d**), KIT (**e**), BRAF (**f**), KRAS (**g**), and NRAS (**h**) in 24,592 human (upper) and 671 canine (lower) tumors. The x-axis indicates amino acid position in the human protein. The y-axis indicates the number of samples with the mutations. The most prominent mutational hotspots are labeled, with the precise human and canine protein position indicated.

We identified 18 canine mutational hotspots (Table S5), which are more likely to harbor cancer drivers^44,45^ and anti-cancer targets. Many of the mutational hotspots are in oncogenes including *PIK3CA*, *KRAS*, *NRAS*, *BRAF*, *KIT*, *ERBB2*, and *EGFR*, as well as in the tumor suppressor *TP53* (Fig. 7; Table S5).

We compared these canine mutational hotspots to those reported in 24,592 human tumors^44,46^. The two species share many mutational hotspots, e.g., *PIK3CA* H1047, *KRAS* G12, *NRAS* Q61, and *BRAF* V600 in human or V588 in canine (Fig. 7). However, species-specific mutational hotspots are also identified, including *PIK3CA* E542/E545 and *ERBB2* S310 in humans, as well as *ERBB2* V659 in dogs (Fig. 7).

*KRAS* and *NRAS* have the same amino acid sequences between the dog and the human. Importantly, the same as in human tumors, the majority of *KRAS* mutations identified in canine tumors are located at mutational hotspot G12, including G12A (detected in 2 melanomas), G12V (in 2 mammary carcinomas), G12D (in 1 mammary carcinoma and 1 squamous cell carcinoma), and G12C (in 1 melanoma) (Table S5). For *NRAS*, all 15 mutations detected in canine tumors are at G13 and G61, both of which are also mutational hotspots in human tumors (Fig. 7). We identified *NRAS* Q61R/K/L mutations in 14 canine tumors, 57% (8/14) of which were hemangiosarcoma, 28% (4/14) malignant melanoma, 7% (1/14) plasma cell tumors and 7% (1/14) soft tissue sarcoma (Table S5).

## Discussion

Cancer genomes harbor actionable gene mutations and clinical sequencing provides immense opportunities for precision medicine in cancer treatment^47,48^. Indeed, clinical sequencing is routinely used in many hospitals in guiding treatment of lung cancers and other cancers in humans. In dogs, clinical sequencing lags significantly behind and is thus underdeveloped for use in cancer treatment. To address this deficiency, we developed the FidoCure^®^ Personalized Genomic Panel, a targeted sequencing panel containing 59 well-known oncogenes and tumor suppressors frequently mutated in human cancer, with common mutational hotspots. Moreover, as clinical sequencing often sequences only the tumor samples due to cost reasons and/or the lack of matching normal samples, we developed an effective germline-somatic mutation discrimination pipeline that maximizes the use of tumor-only sequencing data. We applied the panel and our pipeline to 671 spontaneous canine tumors across 23 tumor types and 96 breeds common to pet dogs in the US. This study, to our knowledge, represents the largest sequencing study of canine tumors to date, and includes tumor types for which mutations have not previously been characterized (e.g., anal sac carcinoma and neuroendocrine carcinoma), or have been less characterized (e.g., soft tissue sarcoma, hepatocellular carcinoma, thyroid carcinoma and mast cell tumors). Our study adds to the growing body of canine comparative oncology studies showing genomic similarities between human and canine cancers and specifically evaluates hotspot mutations that can be targeted with a precision medicine approach. Our study provides a much-needed resource in canine cancer research, accelerating canine precision medicine and enhancing the canine model in human cancer research.

Our analysis largely captures the landscape of hotspot mutations in canine tumors, which are similar to the mutational landscapes reported by previous whole exome or genome sequencing studies (e.g., *TP53*, *NRAS* and *PIK3CA* mutations in hemangiosarcoma, *TP53* and *SETD2* mutations in osteosarcoma, *ERBB2* mutation in pulmonary carcinoma, *BRAF* mutation in urothelial carcinoma and *FBXW7* mutation in lymphoma)^18,20,22–27,29,35–38,49,50^. Consistent with previous research^18,20,22–27,29,35–38,49,50^, our results indicated that *TP53* is the most recurrently mutated gene across tumor types. *TP53* mutations are significantly more common in sarcomas than in carcinomas. This may be due to differences in the cell of origin and the mechanisms of tumorigenesis in these cancer types. Carcinomas originate from polarized epithelial cells or their progenitors, and alterations of cell polarity genes and loss of cell polarity are likely the major drivers of accelerated cell proliferation in carcinoma development^51–53^. Sarcomas originate from mesenchymal cells, for which loss of function of *TP53* leads to defective cell cycle checkpoints and accelerated proliferation. Our studies also identified other frequently mutated genes reported in canine cancer, including chromatin modeling genes (*ARID1A, KMT2D* and others).

Our study finds *TP53* mutated in ~ 46% osteosarcomas, lower than those reported by several publications^23,54^. One reason for this discrepancy is that we examined only somatic base substitutions and small indels, not including somatic copy number alterations, unlike the other studies^23,25^. Moreover, we only sequenced the exons of *TP53*, not the entire gene, and hence were unable to identify intronic translocations and other aberrations found by whole genome sequencing (WGS)^25^. Indeed, Gardner et al.^25^ reports that WGS found *TP53* mutated in 71% cases, whereas whole exome sequencing (WES) found *TP53* muted in only 38% cases. Second, the sample size of our study (46 cases) and other publications (26–66)^25,25,54^ is not large enough to represent the population, resulting in variations among the studies. Similarly, our study identifies *BRAF* V588E (equivalent to V600E in humans) in 35% urothelial carcinomas, lower than several publications^9,19,55,56^. Again, the small sample size (20–66 cases) is a reason for the discrepancy. Another reason is the approach. The cited publications used Sanger sequencing^9,19^, restriction fragment length polymorphism genotyping^19^, or droplet digital PCR^55^ to specifically target the mutation. These methods may be more sensitive and/or have a higher false positive rate, compared to deep sequencing strategies like ours. Indeed, Cronise et al.^56^ reports that WES identified the mutation in 36% cases, while targeted Sanger sequencing identified the mutation in 70% cases.

Mutational landscape varies by tumor type but is largely breed-independent, consistent with a previous pan-cancer and pan-breed study that investigated whole exome data from 591 canine tumors^38^. The largest difference is between carcinomas and sarcomas, with significant differences found in the mutational frequency of *TP53*, *PIK3CA*, *NRAS*, *ERBB2* and *BRAF*. Carcinomas are more variable than sarcomas in their mutational spectrum and mutational burden. Pulmonary adenocarcinoma and squamous cell carcinoma are highly enriched in *BRAF* and *TP53* mutations respectively, and have the highest mutation burden among the tumor types investigated. Anal sac carcinoma and thyroid carcinoma, however, are depleted in *TP53* mutation and mutations in other prominent genes, and have lower mutation burden. The few mutations detected in canine thyroid carcinomas occur in genes including *BRAF* and *KRAS,* both of which are frequently mutated in human thyroid carcinoma^58^.

We acknowledge that the lack of mutations does not mean that the genes are not altered. For example, ERBB2 mutation is found in only one case of anal sac carcinoma despite the overexpression of this receptor identified in 80% of cases in a previous study^57^. Differences in gene expression and mutational profile were also seen in urothelial carcinoma, which is characterized by overexpression of *EGFR* and *ERBB2* in approximately 70% and 60% of the tumors, respectively, and clinical response to EGFR/ERBB2 inhibitors. However, mutations are not identified in either gene in our study or research by others^59–61^. Thus, further studies are needed to comprehensively identify alterations in these tumors.

Our study finds that *NRAS* mutations are mutually exclusive with *TP53* mutations in splenic hemangiosarcoma, reaffirming the existence of different molecular subtypes of the same histology type^27^. Interestingly, *NRAS* mutations were not identified in non-splenic hemangiosarcoma, but mutations (particularly Q61R) in this gene are commonly seen in the splenic form^27^. This is the first time a statistically significant difference in genomic profiles of different anatomic locations of hemangiosarcoma has been reported, and may help guide therapeutic strategies.

Our work reveals that numerous mutational hotspots are shared between dogs and humans, including *PIK3CA* H1047, *BRAF* V600/V588, *KRAS* G12 and others. These findings further position dogs as a powerful translational model for human and veterinary oncology, as both existing and novel targeted therapies for these mutations (e.g., PIQRAY for *PIK3CA*^62^, PLX4032 for *BRAF* V600E mutations^63^) can be assessed in canine cancer patients. Among 20 canine hotspots identified, 13 overlap with those of human cancer. Mutations in five oncogenes are identified as hotspots, representing a unique opportunity to apply targeted therapy translated from human experience.

Our analysis also revealed species-specific mutational hotspots, including *PIK3CA* E545/2K mutations found only in human cancers. *ERBB2* V659EE and *TP53* R209H/C, *KIT* L575P, *KRAS* Q61H/K/R are identified as canine-only hotspots. Further studies are needed to better understand the mechanisms underlying these differences, which will assist anti-cancer drug development and precision medicine in both species.

The FidoCure^®^ Personalized Genomic Panel and our somatic-germline mutation discrimination pipeline effectively capture the landscape of actionable hotspot mutations in canine tumors. We anticipate that this resource will accelerate canine cancer genomic research, significantly increasing the use of the canine model in precision medicine and anti-cancer drug development for both humans and dogs.

## Materials and methods

### Ethical statement

This study was performed in accordance with a protocol approved by the Institutional Ethics Committee of the One Health Company. Prior to enrollment, pet-owners were required to sign an informed consent. No additional procedures were performed on client owned dogs thus this trial does not fall under any regulations overseeing experimental animal trials.

### Enrollment and sample collection

Client-owned dogs with histologically confirmed cancer diagnoses were enrolled in FidoCure^®^ by 200 veterinarians in clinical practice. A total of 671 individual biopsies taken from May of 2019 until September of 2020 were analyzed through the FidoCure^®^ Precision Medicine Platform, the proprietary name of The One Health Company’s precision medicine unit. Upon enrollment, tissue re-cuts obtained from formalin-fixed paraffin embedded (FFPE) tumor tissue used for histopathologic diagnosis were requested from the appropriate veterinary diagnostic laboratory. These tissues were evaluated by practicing board-certified veterinary pathologists and only tissue confirmed to be neoplastic progressed to genomic sequencing.

### Library preparation and next generation sequencing

Genomic DNA (gDNA) was extracted from FFPE tissues using the Mag-Bind^®^ FFPE DNA/RNA kit (Omega Bio-tek). The quality of the extracted gDNA was confirmed using the Agilent Genomic DNA ScreenTape Assay (Agilent) and the amount of gDNA was quantified using the Qubit dsDNA HS assay kit (Thermo Fisher). DNA samples with a major peak of 2000 bp and more than 30% of fragments being > 500 bp were chosen for sequencing. The DNA library was constructed using the SureSelect Low Input library prep kit (Agilent) according to the manufacturer's protocol.

The FidoCure^®^ Precision Medicine Platform targets the coding exons of the genes *ABL1*, *ALK*, *APC*, *ARID1A*, *ATM*, *BCL2*, *BCL6*, *BRAF*, *BRCA1*, *BRCA2*, *BTK*, *CDK2*, *CDK4*, *CDK6*, *CDKN2A*, *CREBBP*, *EGFR*, *ERBB2*, *FBXW7*, *FGFR1*, *FGFR2*, *FGFR3*, *FLT1/VEGFR1*, *FLT3*, *FLT4/VEGFR3*, *HDAC1*, *HIF1*, *HNF1*, *HRAS*, *JAK1*, *JAK2*, *JAK3*, *KDR/VEGFR2*, *KIT*, *KMT2C*, *KMT2D*, *KRAS*, *MEK/MAP2K1*, *MET*, *mTOR*, *NF1*, *NOTCH1*, *NRAS*, *TP53*, *PARP1*, *PDGFRa*, *PDGFRβ*, *PIK3CA*, *PTEN*, *PTPRD*, *PTPRT*, *RAF1*, *RB1*, *RET*, *ROS1*, *SETD2*, *SMAD4*, *SMARCA4*, and *TERT*. These genes are commonly mutated in human cancers and targeted by commercially available oncology panels.

Hybrid capture-based enrichment of the targeted genes was performed using the SureSelect custom DNA Target Enrichment Probes and SureSelect XT Hyb and Wash kit following manufacturer’s instructions. The final library was quantified using qPCR and pooled for sequencing on the Illumina^®^ platform (Illumina, California, USA) with a read length configuration of 150 paired-end (PE) for up to 6M PE reads (3M in each direction), yielding target read depth averaging at 500 × and with a distribution shown in Fig. S2. Sequencing was performed in a CLIA-certified CAP-accredited laboratory.

### Variant calling and evaluation

The sequence read pairs were mapped to the canine reference genome (CanFam3.1)^43^ using BWA^64^ (version 0.7.17). Concordantly and uniquely mapped pairs with at least one read with ≥ 1 bp overlapping a coding sequence (CDS) region of the canFam3 1.99 GTF annotation were used to find mutations. Germline base substitutions and small indels were first called by applying GATK^65^ 3.8.1 HaplotypeCaller to the bam files of individual tumor or normal samples with parameters of dontUseSoftClippedBases and -stand_call_conf 20.0. Variants were then filtered with GATK VariantFiltration with parameters of FS > 30.0 and QD < 2.0. Furthermore, variants with total read coverage < 10 were excluded.

### Tumor mutation burden (TMB) calculation

TMB value was calculated by $TMB = \frac{total\;somatic\;mutations}{{total\;callable\;bases\;in\;million}}$ for each tumor, where the “total somatic mutations” are the sum of somatic mutations indicated in Table S2 for the tumor. Callable bases were identified by applying the GATK CallableLoci function to the realigned and duplicate-removed bam file of the tumor, with a minimum base quality set to 10. Samples with a very small number of callable bases (< 11,143) were excluded from TMB calculation.

### Germline-somatic mutation discrimination

Mutations identified above were subjected to our germline-somatic mutation discrimination pipeline outlined in Fig. 2. First, these mutations were compared to > 9M known germline mutations collected from databases and literature^38,40–43^ to identify and filter out germline mutations. Then, the remaining were divided into groups. For mutations found in ≥ 5 dogs, those with a VAF distribution clustered around 50% or near 100% were classified as heterozygous or homozygous germline mutations respectively. For mutations found in < 5 dogs, those whose human counterparts have been reported to be somatic mutations in human cancers were considered somatic mutations.

Jensen–Shannon (JS) divergence was calculated between two distributions using R package philentropy (version 0.7.0). For permutation testing, the two distributions of interest were combined, random samples of the same size as the original groups were taken, and the JS divergence was calculated between the random samples. This was repeated 100,000 times. The proportion of JS divergences from random samples greater than the original observed JS divergence was considered the p-value.

Somatic mutations identified above were then annotated with Annovar^66^ (version 2017Jul16).

### Definition of somatic mutational hotspots

Mutational hotspots in each species were annotated using the method developed by Chang et al*.*^44^, by identifying positions mutated more frequently than the background mutation rate with a cutoff of recurrence in ≥ 4 samples. Mutations at different nucleotide positions in the same codon of a gene and different nonsynonymous and synonymous base substitutions in the same codon were considered together.

### Statistics and reproducibility

Statistical analyses were performed using R (version 4.1.0)^67^. Fisher’s exact tests were used to compare mutation-positive and mutation-negative groups with categorical features to identify enrichment or depletion of variants in different categories. Multiple testing correction was applied using the Benjamini–Hochberg method to obtain the adjusted p values. For all tests, a two-sided adjusted p-value of < 0.05 was considered statistically significant. Enrichment scores were determined by − log10(adjusted p), with positive values indicating enrichment and negative values indicating depletion.

## Supplementary Information

Supplementary Table S1.

Supplementary Table S2.

Supplementary Table S3.

Supplementary Table S4.

Supplementary Table S5.

Supplementary Figure S1.

Supplementary Figure S2.

## Data Availability

All original sequencing data used for the analysis were submitted to the Sequence Read Archive (SRA) database under the Accession ID of PRJNA880901.

## Code availability

All original codes used in this study, including those for germline-somatic mutation discrimination, are freely available on GitHub at https://github.com/ZhaoS-Lab/Mutation (10.5281/zenodo.6991330).

## References

[1] DiMasi JA, Grabowski HG, Hansen RW (2016). Innovation in the pharmaceutical industry: New estimates of R&D costs. J. Health Econ..

[2] Van Norman GA (2016). Drugs, devices, and the FDA: Part 1. JACC Basic Transl. Sci..

[3] Van Norman GA (2019). Phase II trials in drug development and adaptive trial design. JACC Basic Transl. Sci..

[4] Paoloni MC, Khanna C (2007). Comparative oncology today. Vet. Clin. North Am. Small Anim. Pract..

[5] MacEwen EG (1990). Spontaneous tumors in dogs and cats: Models for the study of cancer biology and treatment. Cancer Metastasis Rev..

[6] Vail DM, MacEwen EG (2000). Spontaneously occurring tumors of companion animals as models for human cancer. Cancer Investig..

[7] Hahn KA, Bravo L, Adams WH, Frazier DL (1994). Naturally occurring tumors in dogs as comparative models for cancer therapy research. In Vivo.

[8] Paoloni M (2009). Canine tumor cross-species genomics uncovers targets linked to osteosarcoma progression. BMC Genom..

[9] Mochizuki H, Breen M (2015). Comparative aspects of BRAF mutations in canine cancers. Vet. Sci. China.

[10] Schiffman JD, Breen M (2015). Comparative oncology: What dogs and other species can teach us about humans with cancer. Philos. Trans. R. Soc. Lond. B Biol. Sci..

[11] Gardner HL, Fenger JM, London CA (2016). Dogs as a model for cancer. Annu. Rev. Anim. Biosci..

[12] Sultan F, Ganaie BA (2018). Comparative oncology: Integrating human and veterinary medicine. Open Vet. J..

[13] Sommer BC, Dhawan D, Ratliff TL, Knapp DW (2018). Naturally-occurring canine invasive urothelial carcinoma: A model for emerging therapies. Bladder Cancer.

[14] Dow S (2019). A role for dogs in advancing cancer immunotherapy research. Front. Immunol..

[15] Paoloni M (2014). Prospective molecular profiling of canine cancers provides a clinically relevant comparative model for evaluating personalized medicine (PMed) trials. PLoS One.

[16] Katogiritis A, Khanna C (2019). Towards the delivery of precision veterinary cancer medicine. Vet. Clin. North Am. Small Anim. Pract..

[17] Bushell KR (2015). Genetic inactivation of TRAF3 in canine and human B-cell lymphoma. Blood.

[18] Elvers I (2015). Exome sequencing of lymphomas from three dog breeds reveals somatic mutation patterns reflecting genetic background. Genome Res..

[19] Decker B (2015). Homologous mutation to human BRAF V600E is common in naturally occurring canine bladder cancer-evidence for a relevant model system and urine-based diagnostic test. Mol. Cancer Res..

[20] Wang G (2017). Actionable mutations in canine hemangiosarcoma. PLoS One.

[21] Ramsey SA (2017). Cross-species analysis of the canine and human bladder cancer transcriptome and exome. Genes Chromosomes Cancer.

[22] Hendricks WPD (2018). Somatic inactivating PTPRJ mutations and dysregulated pathways identified in canine malignant melanoma by integrated comparative genomic analysis. PLoS Genet..

[23] Sakthikumar S (2018). SETD2 is recurrently mutated in whole-exome sequenced canine osteosarcoma. Cancer Res..

[24] Wong K (2019). Cross-species genomic landscape comparison of human mucosal melanoma with canine oral and equine melanoma. Nat. Commun..

[25] Gardner HL (2019). Canine osteosarcoma genome sequencing identifies recurrent mutations in DMD and the histone methyltransferase gene SETD2. Commun. Biol..

[26] Megquier K (2019). Comparative genomics reveals shared mutational landscape in canine hemangiosarcoma and human angiosarcoma. Mol. Cancer Res..

[27] Wang G (2020). Molecular subtypes in canine hemangiosarcoma reveal similarities with human angiosarcoma. PLoS One.

[28] Wong S (2020). Genomic landscapes of canine splenic angiosarcoma (hemangiosarcoma) contain extensive heterogeneity within and between patients. bioRxiv.

[29] Amin SB (2020). Comparative molecular life history of spontaneous canine and human gliomas. Cancer Cell.

[30] Wong K (2021). Comparison of the oncogenomic landscape of canine and feline hemangiosarcoma shows novel parallels with human angiosarcoma. Dis. Model. Mech..

[31] Gordon I, Paoloni M, Mazcko C, Khanna C (2009). The Comparative Oncology Trials Consortium: Using spontaneously occurring cancers in dogs to inform the cancer drug development pathway. PLoS Med..

[32] Davis BW, Ostrander EA (2014). Domestic dogs and cancer research: A breed-based genomics approach. ILAR J..

[33] McDonald JT (2018). Comparative oncology DNA sequencing of canine T cell lymphoma via human hotspot panel. Oncotarget.

[34] Kim JH, Megquier K, Thomas R, Sarver AL (2021). Genomically complex human angiosarcoma and canine hemangiosarcoma establish convergent angiogenic transcriptional programs driven by novel gene. Mol. Cancer Res..

[35] Liu D (2014). Molecular homology and difference between spontaneous canine mammary cancer and human breast cancer. Cancer Res..

[36] Lee K-H, Hwang H-J, Noh HJ, Shin T-J, Cho J-Y (2019). Somatic mutation of PIK3CA (H1047R) is a common driver mutation hotspot in canine mammary tumors as well as human breast cancers. Cancers.

[37] Kim T-M (2020). Cross-species oncogenic signatures of breast cancer in canine mammary tumors. Nat. Commun..

[38] Alsaihati BA (2021). Canine tumor mutational burden is correlated with TP53 mutation across tumor types and breeds. Nat. Commun..

[39] Alexandrov LB (2020). The repertoire of mutational signatures in human cancer. Nature.

[40] Plassais J (2019). Whole genome sequencing of canids reveals genomic regions under selection and variants influencing morphology. Nat. Commun..

[41] Hunt SE (2018). Ensembl variation resources. Database.

[42] Bai B (2015). DoGSD: The dog and wolf genome SNP database. Nucleic Acids Res..

[43] Hoeppner MP (2014). An improved canine genome and a comprehensive catalogue of coding genes and non-coding transcripts. PLoS One.

[44] Chang MT (2016). Identifying recurrent mutations in cancer reveals widespread lineage diversity and mutational specificity. Nat. Biotechnol..

[45] Trevino V (2020). HotSpotAnnotations—A database for hotspot mutations and annotations in cancer. Database.

[46] Chang MT (2018). Accelerating discovery of functional mutant alleles in cancer. Cancer Discov..

[47] Wu L (2022). Landscape of somatic alterations in large-scale solid tumors from an Asian population. Nat. Commun..

[48] Zehir A (2017). Mutational landscape of metastatic cancer revealed from prospective clinical sequencing of 10,000 patients. Nat. Med..

[49] Wang J (2018). Collaborating genomic, transcriptomic and microbiomic alterations lead to canine extreme intestinal polyposis. Oncotarget.

[50] Wang J (2019). Proliferative and Invasive Colorectal Tumors in Pet Dogs Provide Unique Insights into Human Colorectal Cancer.

[51] Li Y (2014). Cancer driver candidate genes AVL9, DENND5A and NUPL1 contribute to MDCK cystogenesis. Oncoscience.

[52] Tang J (2014). Cancer driver–passenger distinction via sporadic human and dog cancer comparison: A proof-of-principle study with colorectal cancer. Oncogene.

[53] Wang T (2020). A qualitative change in the transcriptome occurs after the first cell cycle and coincides with lumen establishment during MDCKII cystogenesis. iScience.

[54] Das S (2021). Immune pathways and TP53 missense mutations are associated with longer survival in canine osteosarcoma. Commun. Biol..

[55] Mochizuki H, Shapiro SG, Breen M (2015). Detection of BRAF mutation in urine DNA as a molecular diagnostic for canine urothelial and prostatic carcinoma. PLoS One.

[56] Cronise KE (2022). Characterizing the molecular and immune landscape of canine bladder cancer. Vet. Comp. Oncol..

[57] Yoshimoto S (2019). Detection of human epidermal growth factor receptor 2 overexpression in canine anal sac gland carcinoma. J. Vet. Med. Sci..

[58] Haroon Al Rasheed MR, Xu B (2019). Molecular alterations in thyroid carcinoma. Surg. Pathol. Clin..

[59] Maeda S (2022). Lapatinib as first-line treatment for muscle-invasive urothelial carcinoma in dogs. Sci. Rep..

[60] Hanazono K (2015). Epidermal growth factor receptor expression in canine transitional cell carcinoma. J. Vet. Med. Sci..

[61] Tsuboi M (2019). Assessment of HER2 expression in canine urothelial carcinoma of the urinary bladder. Vet. Pathol..

[62] Fritsch C (2014). Characterization of the novel and specific PI3Kα inhibitor NVP-BYL719 and development of the patient stratification strategy for clinical trials. Mol. Cancer Ther..

[63] Halaban R (2010). PLX4032, a selective BRAF(V600E) kinase inhibitor, activates the ERK pathway and enhances cell migration and proliferation of BRAF melanoma cells. Pigment Cell Melanoma Res..

[64] Li H, Durbin R (2010). Fast and accurate long-read alignment with Burrows–Wheeler transform. Bioinformatics.

[65] Van der Auwera GA (2013). From FastQ data to high confidence variant calls: The Genome Analysis Toolkit best practices pipeline. Curr. Protoc. Bioinform..

[66] Wang K, Li M, Hakonarson H (2010). ANNOVAR: Functional annotation of genetic variants from high-throughput sequencing data. Nucleic Acids Res..

[67] R Development Core Team. R: A language and environment for statistical computing (2023).

