# Supplementary material for: Shared hotspot mutations in oncogenes position dogs as an unparalleled comparative model for precision therapeutics
Source: Sci Rep. 2023 Jul 6;13:10935. doi: 10.1038/s41598-023-37505-2 (PMC10325973; doi:10.1038/s41598-023-37505-2)
Supplement: Supplementary file 7 — Supplementary Figure S2. [file 41598_2023_37505_MOESM7_ESM.pdf]

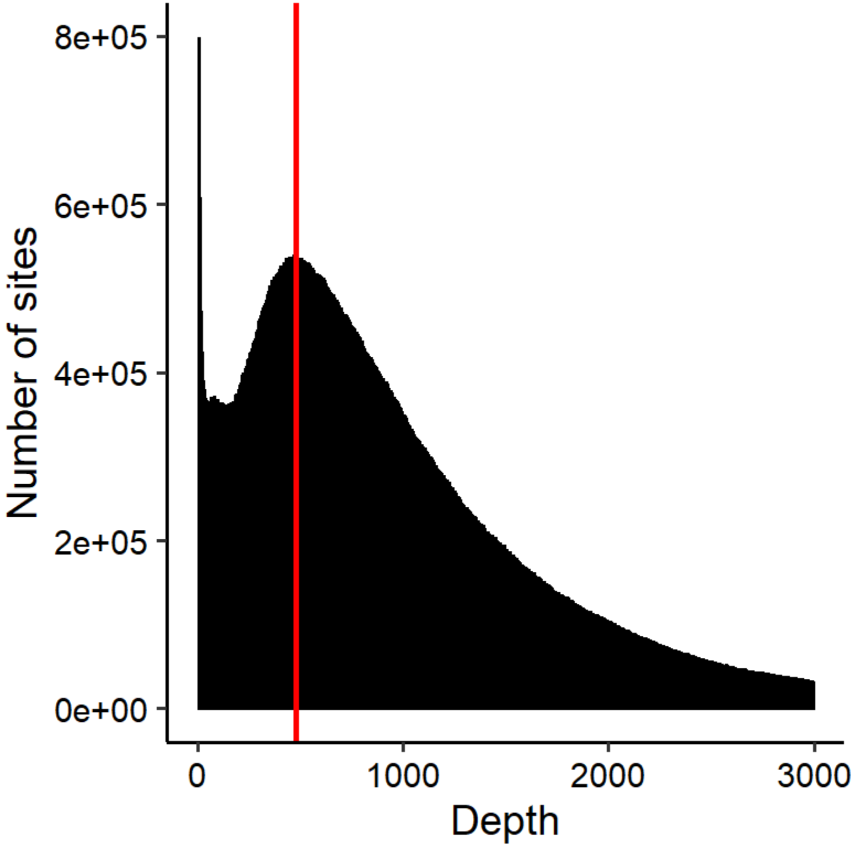

Supplementary Figure 2: The distribution of base coverage at each base pair position of targeted regions (exons) of the 50 genes sequenced.
