# Supplementary material for: Shared hotspot mutations in oncogenes position dogs as an unparalleled comparative model for precision therapeutics
Source: Sci Rep. 2023 Jul 6;13:10935. doi: 10.1038/s41598-023-37505-2 (PMC10325973; doi:10.1038/s41598-023-37505-2)
Supplement: Supplementary file 6 — Supplementary Figure S1. [file 41598_2023_37505_MOESM6_ESM.pdf]

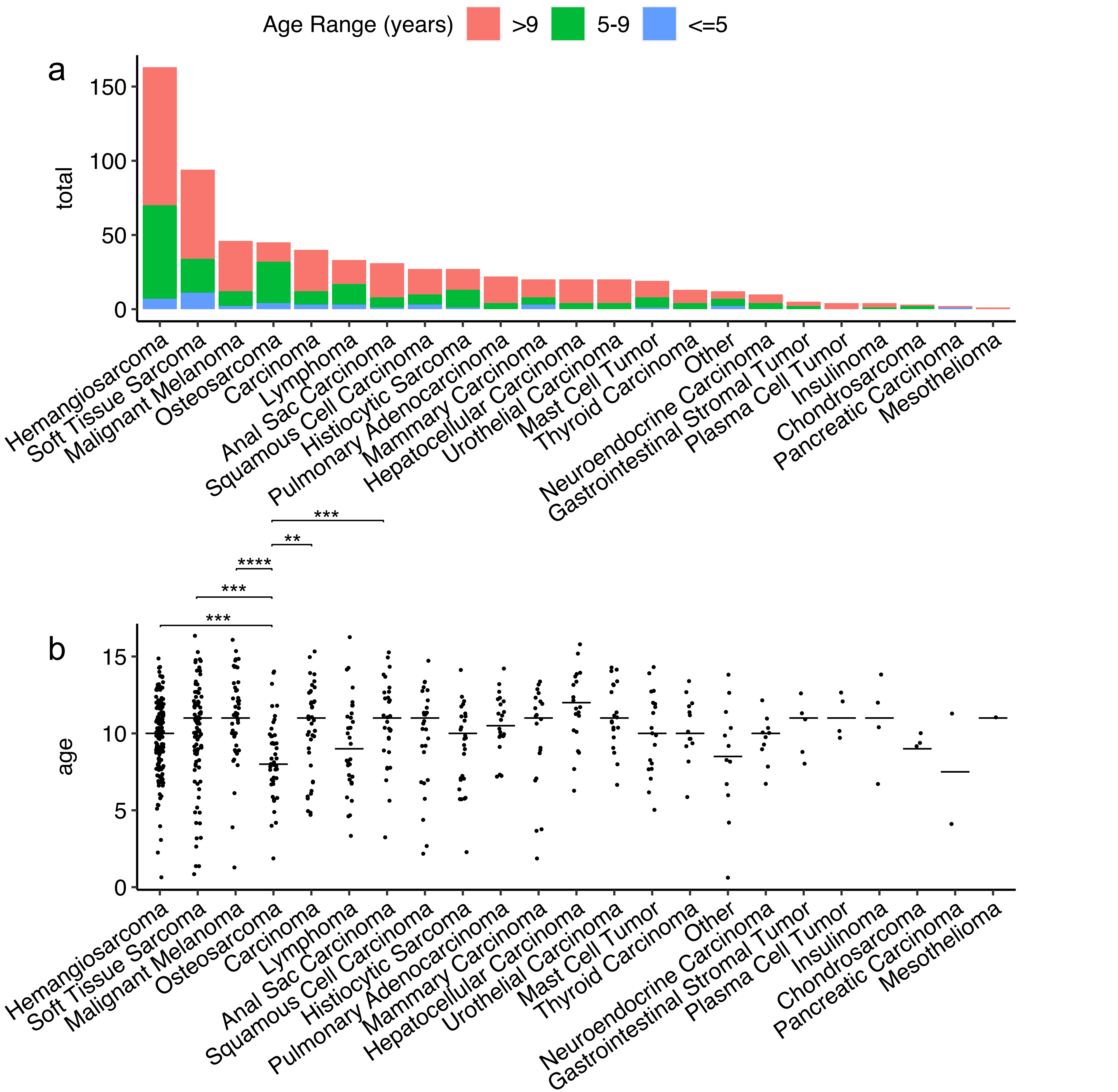

Supplementary Figure 1: Dogs with osteosarcoma are significant younger compared to other tumor types.
a. Number of dogs of each tumor type divided in <= 5 years, 5-9 years and >9 years old. **b** Distribution of age in each tumor type. Each dot represents a sample, while the black horizontal lines indicate the median age in the respective cancer types.
